# RapidEELS: machine learning for denoising and classification in rapid acquisition electron energy loss spectroscopy

**DOI:** 10.1038/s41598-021-97668-8

**Published:** 2021-09-30

**Authors:** Cassandra M. Pate, James L. Hart, Mitra L. Taheri

**Affiliations:** 1 Department of Materials Science & Engineering, Johns Hopkins University, Baltimore, MD 21218 USA; 2 Currently at Department of Mechanical Engineering & Materials Science, Yale University, New Haven, CT 06511 USA

**Keywords:** Materials science, Characterization and analytical techniques

## Abstract

Recent advances in detectors for imaging and spectroscopy have afforded in situ, rapid acquisition of hyperspectral data. While electron energy loss spectroscopy (EELS) data acquisition speeds with electron counting are regularly reaching 400 frames per second with near-zero read noise, signal to noise ratio (SNR) remains a challenge owing to fundamental counting statistics. In order to advance understanding of transient materials phenomena during rapid acquisition EELS, trustworthy analysis of noisy spectra must be demonstrated. In this study, we applied machine learning techniques to denoise high frame rate spectra, benchmarking with slower frame rate “ground truths”. The results provide a foundation for reliable use of low SNR data acquired in rapid, in-situ spectroscopy experiments. Such a tool-set is a first step toward both automation in microscopy as well as use of these methods to interrogate otherwise poorly understood transformations.

## Introduction

Advances in electron imaging and spectroscopy instrumentation now allow for time-resolved data acquisition at rates over 1000 frames per second. Specifically, development of direct detection electron sensors, including both back thinned monolithic active pixel designs and thick hybrid pixel designs, enable counting of individual electron events for shot noise limited data acquisition^1,2^. This led to significant advancement in numerous areas of electron microscopy, including cryo-transmission electron microscopy (TEM)^3^ , low signal to noise ratio (SNR) electron energy loss spectroscopy (EELS) acquisition^4^, in-situ TEM/EELS^5^, and 4D scanning transmission electron microscopy (STEM)^6^. While novel EELS direct detection systems allow for rapid acquisition, this function has not been utilized to its full potential as any increase in speed comes at the expense of the signal-to-noise ratio, or SNR. To understand emerging and evolving phenomena, spectra need to be acquired in as short of timescales as possible to capture transient behavior. At high frame rates, however, the low SNR can render datasets too noisy for analysis (e.g. quantification of oxidation state). In transition metal oxides, for example, many methods used for valence determination, e.g. *L*$_{3}$/*L*$_{2}$ ratio, chemical shift, or O *K*-edge pre-peak analysis can yield contradictory results regardless of SNR. A reduction in SNR degrades spectra as compared to those acquired at long time scales, and in some cases, renders data unusable or untrustworthy. In addition, calibration differences between different machines or the same machine at a different time, can further complicate spectra analysis by introducing translational shifts. Another problem is plural scattering, which leads to thickness dependent spectral changes.

## Background

Machine learning tools present a solution to raise the information limit of rapidly acquired spectra, building a foundation for characterizing and tracking transient phenomena at shorter time scales. Convolutional neural networks (CNN), specifically, have become common in fields with challenges that cannot be overcome by more basic human-driven domain engineering and statistical analysis due the high-dimensionality of the input or a lack of ‘ground truth’, such as speech recognition and natural language processing^7–9^, image classification^10–13^ and feature extractions and classification of electroencephalography signals which tend to have a low SNR and be sensitive to background noise^7,8^. The ‘learned’ lower-dimension information may then be linked to other models, such as classifiers, clustering algorithms, or visualization techniques. Autoencoders (AEC) for image denoising is well established field^14–17^ with convolutional AECs specifically being highly successful even on small datasets^18,19^.

The use of neural networks (NNs) for spectra classification in EELS has been limited, with the majority of NN-based spectra classification having been focused on other techniques, such as Raman or X-ray absorption spectroscopy^12,20–22^ or denoising of STEM images^23–26^. The application of NNs to EELS data has, to date, relied on feature engineering, use of a reference spectrum, and requiring a priori knowledge during post-processing to extract useful information. Linear models have been used for the determination of oxidation states in metal oxides^27^ and manganese valence states^28^. Kalinin et. al explored unmixing and supervised classification on heterogeneous self-assembled monolayer films of doped-semiconductor nanoparticles^29^. Blum et. al proposed a new algorithm for strong metal-support interactions and exploring encapsulation signals in heterogeneous catalysts^30^. Most notably, Chatzidakis et. al^31^ investigated EELS spectra oxidation state classification with a mix of fully dense and fully CNN architectures and samples augmented with varying simulated noise levels. Advances in STEM allows for EELS spectral images of beam-stable materials to be obtained with a high SNR^32,33^. However, the high current density and longer dwell time needed may damage sensitive samples^33–35^.

Autoencoders are a type of unsupervised neural network model that maps high dimensional input features to a lower dimension and then reconstructs the original input from the lower dimensional layer, i.e. the latent space. Generally, AECs have a three-part symmetric structure: encoder, latent space, decoder. The encoder maps high dimension input data to a lower dimension representation. In doing so, it learns and builds patterns in the data that might be overlooked by or invisible to human-designed models or analysis. These patterns are represented in a reduced dimension vector space, also called latent space, that produce a more generalized characterization of the data. A decoder, inverse in structure to the encoder, is then trained to reconstruct the input from the latent space features extracted by the encoder. A variation of this, commonly used in the image reconstruction field, is the semi-supervised denoising auto encoder. The encoder maps a partially destroyed or incomplete input to the latent space, decodes as a non-destroyed (denoised) image, and is trained to minimize the average reconstruction error between the reconstruction and the ground truth image.

In this study, we utilize a dual Autoencoder-Classifier algorithm to denoise low SNR spectra collected at 400FPS, as a starting point for rapid identification of real-time EELS data. The reduction of $\hbox {SrFeO}_{3-\delta }$ ($\delta$ represents the oxygen deficiency) is used as a model experiment, with the detection of oxidation state changes from nominally $\hbox {SrFeO}_{{3}}$ ($\hbox {Fe}^{4+}$) to $\hbox {SrFeO}_{2.5}$ ($\hbox {Fe}^{3+}$) as target metrics to benchmark our algorithms in terms of accuracy and effectiveness. Specifically, we employed a stacked convolutional denoising AEC and fully connected classifier on a model system to study noise reduction and oxidation state classification for low SNR EELS spectra collected at 400FPS. Classification accuracy on the latent space representations indicate how successfully the AEC learns relevant, unique features and can be expanded on in the future for more complex unsupervised applications, or high frame rates studies of non-equilibrium phenomena. The notebooks developed during this study can be found on GitHub (https://github.com/patecm/rapidEELS).

## Methodology

As a model system to investigate EELS fine structure changes, we study the reduction of a $\hbox {SrFeO}_{3-\delta }$ thin film. A $\hbox {SrFeO}_{3-\delta }$ film of roughly 20 nm was grown via molecular beam epitaxy on a ($\hbox {LaAlO}_{3-\delta })_{0.3}$($\hbox {Sr}_{{2}} \hbox {TaAlO}_{6})_{0.7}$ (LSAT) substrate^36^. Post-growth, the film was oxidized with an ozone anneal to produce perovskite $\hbox {SrFeO}_{{3}}$, with $\delta$ $\approx$ 0^37^. For $\delta$ = 0, $\hbox {SrFeO}_{{3}}$ is metallic and paramagnetic with a cubic perovskite structure and Fe is in a formal 4+ oxidation state. With reduction to $\delta$ = 0.5, the brownmillerite $\hbox {SrFeO}_{2.5}$ structure is stabilized, which is an insulating antiferromagnet with Fe in an average 3+ oxidation state^38^. While the metal-to-insulator and magnetic transitions hold technological relevance, we are mainly interested in the core-loss spectral changes associated with the altered Fe oxidation state and Fe-O bond geometry.

STEM-EELS measurements were performed with a JEOL 2100F instrument and a Gatan Imaging Filter Quantum equipped with a K2 direct electron detector operated in counting mode^4^. The STEM convergence semi-angle was 8 mR, and the EELS collection semi-angle was 24 mR. For EELS, the 5 mm aperture was used with a dispersion of 0.125 eV/channel. A focused ion beam (FEI DB235) was used to prepare a conventional lift-out for TEM analysis. Final thinning was performed with 5 keV Ga ions. The FIB lift-out was then re-oxidized via the ozone anneal, assuming some loss of O during FIB sample preparation. After initial TEM and EELS analysis, the lift-out was removed from the TEM and reduced ex situ by annealing on a hot plate at 300 C in ambient atmosphere for 10 min Subsequently, the sample was placed back in the TEM for further TEM and EELS analysis. We note the presence of an interfacial region between the $\hbox {SrFeO}_{3-\delta }$ film and LSAT substrate (present before and after ex situ annealing), characterized by planar defects observed with HRTEM and reduced intensity observed in STEM-ADF (indicating a lower average mass density). This defective interfacial region is excluded from subsequent imaging and spectral analysis.

**Figure 1:**
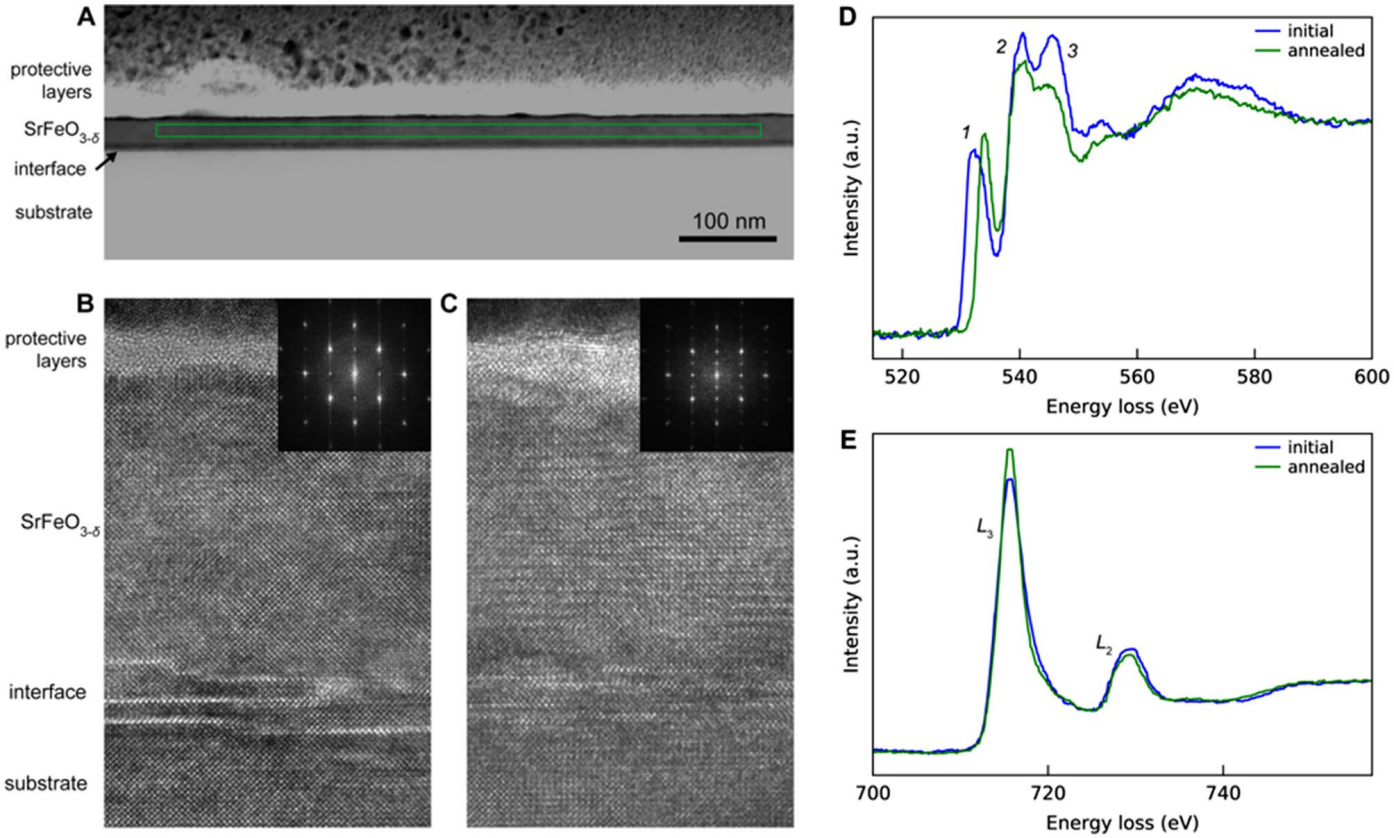
(**A**) STEM ADF of the $\hbox {SrFeO}_{3-\delta }$ film. The green box shows the location of one SI. (**B**, **C**) are HRTEM images of the $\hbox {SrFeO}_{3-\delta }$ before and after the ex situ anneal, respectively. The insets show Fourier transforms (FT) of the $\hbox {SrFeO}_{3-\delta }$ layer, excluding the defective interfacial region. To obtain adequate SNR for the FT of the annealed sample, FTs from multiple HRTEM images were summed together. **D.** and **E.** are the O *K*-edge and the Fe *L*-edge, respectively, comparing the initial and annealed $\hbox {SrFeO}_{3-\delta }$. The data was averaged over 10 200 pixels, with a local thickness of 0.7 MFP. A power-law background subtraction was applied to both edges, and the edges were normalized to their post-edge continuum intensity.

HRTEM imaging and EELS show a clear transition in the $\hbox {SrFeO}_{3-\delta }$ film after annealing. As shown in Fig. 1B, before the ex-situ anneal, the $\hbox {SrFeO}_{3-\delta }$ shows cubic symmetry as expected for perovskite $\hbox {SrFeO}_{{3}}$. After the anneal, half-order peaks are observed in the HR-TEM Fourier transform, indicative of O vacancy ordering in the brownmillerite $\hbox {SrFeO}_{2.5}$ structure. Spectrally, clear changes in both the O *K*-edge and Fe *L*-edge are observed, which are consistent with the perovskite $\hbox {SrFeO}_{{3}}$ to brownmillerite $\hbox {SrFeO}_{2.5}$ transition studied with X-ray absorption spectroscopy at the same edges^39^. Spectral analysis shows that the energy difference between the O *K*-edge onset and the Fe *L*-edge onset is reduced from 183.1 to 181.5 eV after annealing, and additionally, the Fe *L*$_{3}$/*L*$_{2}$ white line ratio increased from 3.8 to 4.6. Both changes are consistent with a nominal decrease in the Fe oxidation state from $\sim$4+ to $\sim$3+ with annealing. Thus, while the precise value of $\delta$ in our film is not known, we conclude a clear transition was induced via the ex-situ anneal, and that the initial and annealed states roughly corresponding to $\hbox {SrFeO}_{{3}}$ and $\hbox {SrFeO}_{2.5}$.

Experimental EELS datasets were used to develop our algorithm. For the initial-state EELS data acquisition, multi-frame spectra images (SIs) were acquired with a pixel size of 1 $\hbox {nm}^2$, 0.0025 s dwell time, and lateral dimensions of 10 638 pixels. Three frames were acquired, and a drift correction was performed between each frame. The SI was vertically aligned within the $\hbox {SrFeO}_{3-\delta }$ layer to only probe the central region of the film, thereby excluding the defective interfacial layer as well as damage at the top of the film (the green box in Fig. 1A shows one representative SI area). Four multi-frame SIs were acquired in the initial-state. Each SI was shifted laterally along the SFO/LSAT interface, which, owing to the sample geometry and FIB preparation method, resulted in sampling various thicknesses of the SFO film. Based on low-loss EELS measurements, the four SIs covered a continuous thickness range from 0.6 to 1.3 inelastic mean free paths (MFPs). Note that the 4 multi-frame SIs give a total of 12 SIs. The exact same procedure was repeated after annealing the sample ex situ. Various irregularities in the protective layers above the $\hbox {SrFeO}_{3-\delta }$ film were used to correctly position the annealed-state SIs with the corresponding initial-state SIs.

**Figure 2:**
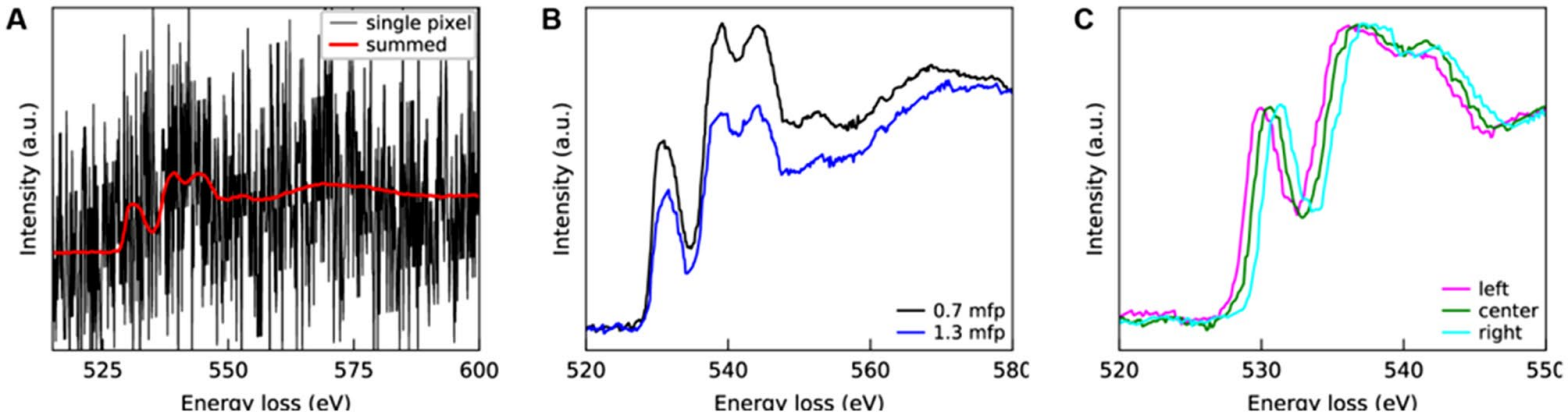
(**A**) Comparison of mean versus single pixel, centered at the same region of the sample. The single pixel values were multiplied by 4230 for comparison. (**B**) Effect of thickness of the initial state O *K*-edge. (**C**) Effect of spatial position of the STEM probe on the EELS energy shift. Scanning the STEM probe across the  600 nm SI box results in an energy shift of  1.5 eV. A power-law background subtraction was performed on all edges.

While the spectral differences associated with the $\hbox {SrFeO}_{{3}}$ to $\hbox {SrFeO}_{2.5}$ transition appear obvious in the data shown in Fig. 1, we note three experimental difficulties which make practical classification of spectra a challenging task. First, the SNR of spectra associated with individual pixel elements is extremely low. For each individual spectra, the average number of electron counts per channel at the O *K*-edge is $\approx$ 15 (for a local thickness of 0.8 MFP), which gives a shot noise limited SNR of sqr(N) = 3.8. This low SNR is shown in Fig. 2A. Secondly, given the large range of sample thicknesses, plural scattering leads to significant spectral changes as a function of thickness. This is shown in Fig. 2B, which compares a thin versus thick region of the SFO in the initial state. Lastly, as the STEM beam scans across the sample, there is an associated shift of the EELS data in energy space. This artifact is shown in Fig. 2C, which compares spectra from the leftmost, central, and rightmost regions of a SI acquired from the annealed-state. Note that from the perspective of the classifier, the effects of thickness (plural scattering) and artificial energy shifts may be considered as additional ‘noise’ sources, which the classifier must deal with to accurately label spectra as ‘initial’ or ‘annealed’. However, from the perspective of the AEC, the effects of thickness and energy shifts on a given spectra should be preserved. The AEC is useless unless it preserves subtle features of interest within a given spectra. Of course, artificial energy shifts are not ‘of interest’ but they may act as a criteria on which to assess the AEC performance.

Training data was grouped into four categories for comparison purposes, based on thickness-based spectral changes and oxidation state. 12 SIs are initial ($\hbox {SrFeO}_{{3}}$) state and 12 have been annealed to $\hbox {SrFeO}_{2.5}$ using the methodology previous described. Each oxidation state contained six “thin” datasets, with a MFP between 0.65 and 0.80, and six “thick” datasets, with a MFP of 0.83–1.33. Hence, the four categories are thin initial, thick initial, thin annealed, and thick annealed, and each category contains 6 SIs. EELS data is collected in a 3D cube, where each 2D pixel of the TEM image has an associated energy spectrum. Each of the twenty-four EELS SIs produced STEM images with 638 by 10 spatial pixels, for a total of 153,120 input spectra. Of these, four SIs were withheld for testing, one from each of the four main categories: thin initial, thick initial, thin annealed, thick annealed. Twenty percent of the remaining training input spectra were used as a validation set.

To facilitate real-time classification of spectra in the future, pre-processing for the ML framework was kept as minimal as possible. Training spectra were imported from dm4 files and cropped from 520 to 580 eV around the O *K*-edge using the HyperSpy package^40^, and binned from 0.125 to 0.25 eV/channel, resulting in 240-feature long spectra. Spectra were normalized from 0 to 1, without any background subtraction. Five-fold cross-validation was performed prior to model training to select the dimensions of the latent space representation, learning rate, percent dropout, training batch-size, and number of training epochs. Mean centering standardization was not performed on the training data, as it was also determined in cross-validation to be unnecessary and in some cases to degrade the reconstruction. Translational shifts between scans or varying sample thicknesses were not corrected for.

**Figure 3:**
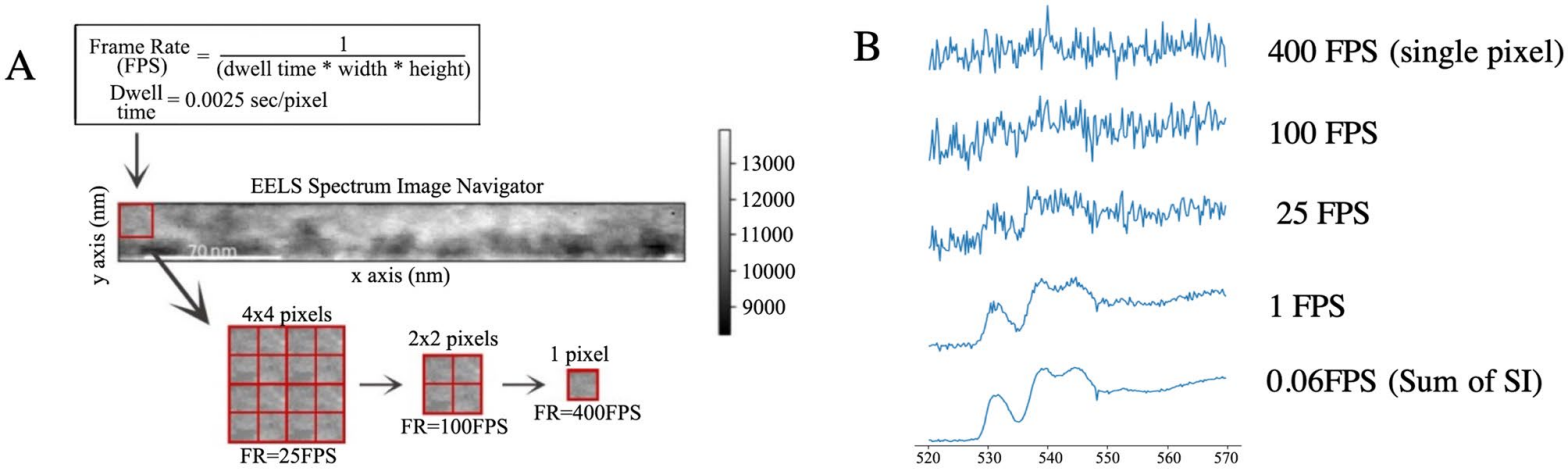
Illustration of the relationship between the (**A**) region of interest (ROI) and frame rate, and the (**B** )impact on the signal to noise ratio when summing all spectra within the ROI. At high frame rates, the low signal to noise ratio makes it impossible to visually analyze or assign structure, as the signal is mostly noise.

To train the AEC toward denoising, ‘ground truths’ were created with a convolution filter across the spatial dimensions of the raw input spectra. Because the oxidation state was uniform across each EELS sample, convolution simulates a lower frame rate and therefore less noisy spectra. A simulated frame rate of 1FPS (filter size 10x40) was chosen since it was shown to produce spectra where oxidation states are visually distinguishable from one another. Fig.  3B shows the difference in the SNR for different simulated frame rates. In contrast to most denoising AEC, where the ground truth is corrupted by adding Gaussian noise, the spectra collected at 400FPS at each pixel was used as the noisy data. The addition of noise has been shown to improve reconstructions and prevent overfitting in denoising AECs^41–43^. Because the noise intrinsic to electron exposure (shot noise) follows a Poisson distribution^44^, Poisson noise was added to the input instead of the more commonly used random noise. PowerLaw background subtraction was performed only on the “ground truth” spectra before normalizing from zero to one for input to the AEC. Thus, the AEC is also trained to perform a background subtraction on the input data, which allows easier analysis on the denoised spectra.

**Figure 4:**
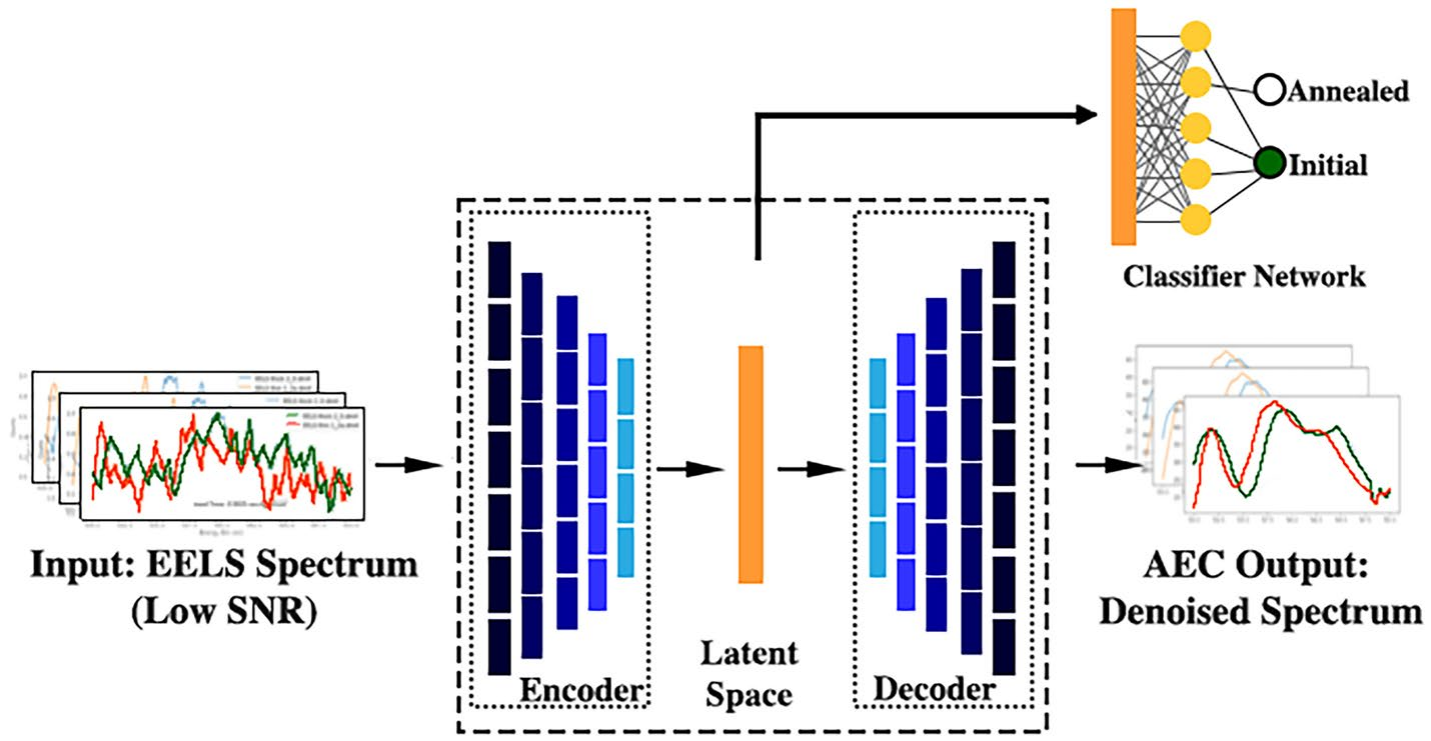
Visualization of the Neural Networks. The autoencoder learns a lower dimensional representation (latent space) of the input data and constructs denoised spectrum as output. The latent space representation of the ‘noisy’ spectrum is passed to the classifier, which determines if the input is initial or annealed state.

The encoder architecture of the AEC consisted of five one-dimensional (1D) convolution hidden layers, which increase in filter size but decrease in kernel size. An overview of the model can be found in Fig. 4 with more details in Table 1 of the Supplemental Information. For some AEC applications, Dropout has proven more effective at preventing over-fitting than other regularization methods such as L1 norm^45–48^.During framework development, Dropout was found to produce better results than L1 regularization. Therefore, a twenty percent Dropout layer between layers is included, with the last convolution layer of the encoder also having a twenty percent dropout bottleneck. Pooling was not used between layers to avoid discarding finer details and features for the reconstructions^49^. Each hidden convolution layer had ReLU activation with the exception of the output layer of decoder, which used a linear activation. The decoder was structured as the inverse of the encoder, but without dropout between layers to preserve the reconstruction of the latent space. Connecting the encoder to the decoder is the latent space, which learns a five-dimension representation of the spectra. The model was fit using the Adam optimizer and minimizing the reconstruction mean square error (MSE) loss function. MSE was chosen over mean absolute error (MAE) because it is more sensitve to outliers^50^ and after demonstrating a higher overall MSE for denoised spectra during cross-validation.

**Figure 5:**
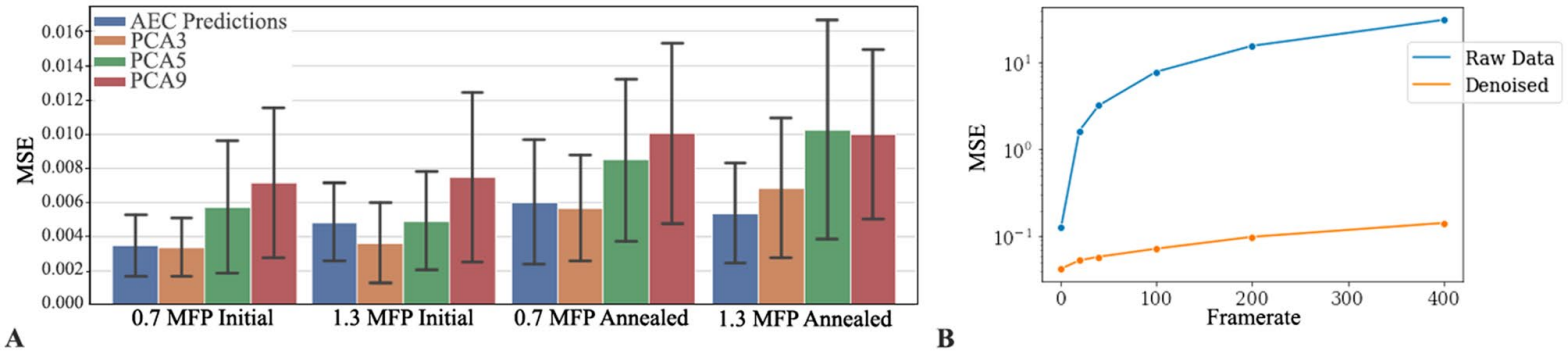
(**A**) Comparison of mean squared error (MSE) between three different PCA reconstructions and the AEC reconstructions, sorted by type of SI in the test dataset. MSE was calculated by comparing the corresponding Ground Truth spectra. (**B**) MSE versus frame-rate for raw denoised spectra.

Post-Autoencoder training, encoder layers were frozen before training the classifier. The latent space containing features extracted by the Encoder were used as input to the classifier, which consist of a single, two neuron dense layer with Softmax activation. All models were trained for 500 epochs each on a NVIDIA GTX1080Ti GPU, with CUDA v10.1 and cuDNN v7.6, using Keras and Tensorflow 2.3.0 back-end. Total training time was approximately 20 min (Fig. 5).

## Results and discussion

**Figure 6:**
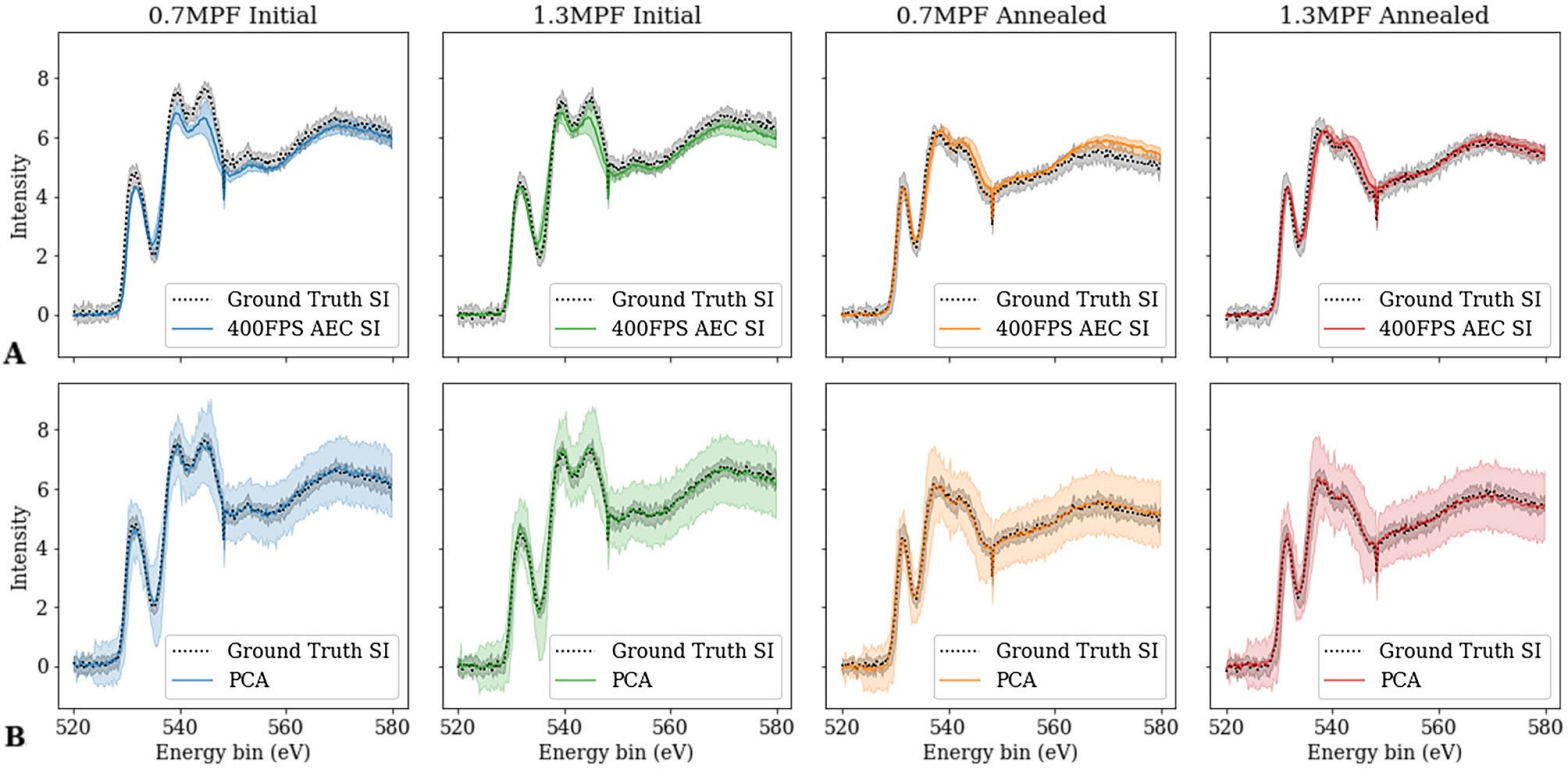
Visual comparison of the four test datasets, at varying oxidation state and sample thickness. Solid lines are the average of the SI and corresponding shaded region is the standard deviation. (**A**) Average of AEC denoised 400FPS spectra (blue, green, orange, red solid lines) in a 40 by 10 pixel section. Corresponding Ground Truth spectra are superimposed with a gray dashed line. (**B**) Average 3 component PCA reconstruction of 400FPS spectra (blue, green, orange, red solid lines) in a 40 by 10 pixel section. Correspond Ground Truth spectra are superimposed with a gray dashed line.

Testing data consisted of four SIs, one of each oxidation state and thickness range, for a total of 25520 spectra. The training data was prepared in the same manner as previously described for the test dataset but without the addition of noise. Additional background subtraction was not performed on the predictions, as the “ground truth” training data had background removed prior to training the autoencoder which results in denoised spectra that are background subtracted. Two key concerns addressed by this study were if fine details and subtle features were maintained in the denoised reconstruction spectra and how well the autoencoder performed compared to common denoising techniques. Principle component analysis (PCA) was selected to benchmark fine feature reconstruction. While commonly used as a dimensionality reduction technique, PCA is also used in the computer vision field as a noise reduction technique^51,52^. To establish general performance, Gaussian curves were fit to the pre-peak and *K*-edge peaks for each denoised pixel result (see Figure 1 of the Supplemental Information), using a least squared approximation to minimize the sum of squared residuals. Gaussian curves were also fit to the pre-peak and Oxygen *K*-edge of the Ground Truth spectra that corresponded to each pixel. Reconstruction errors of each sample class were determined by mean square error (MSE). Visualizations of the PCA reconstructions and AEC densoised SI are shown in Fig. 6. While the AEC denoised spectra had a lower MSE than the 5 and 7 component PCA reconstructions, 3 component PCA reconstruction had a comparable or lower MSE than the AEC denoised spectra, with the exception of the thicker annealed samples. The changes in peak position and height of the pre-peak and *K*-edge from the Ground Truth pixels, after Gaussian fitting, are shown in Table 2 of the Supplemental Information.

**Figure 7:**
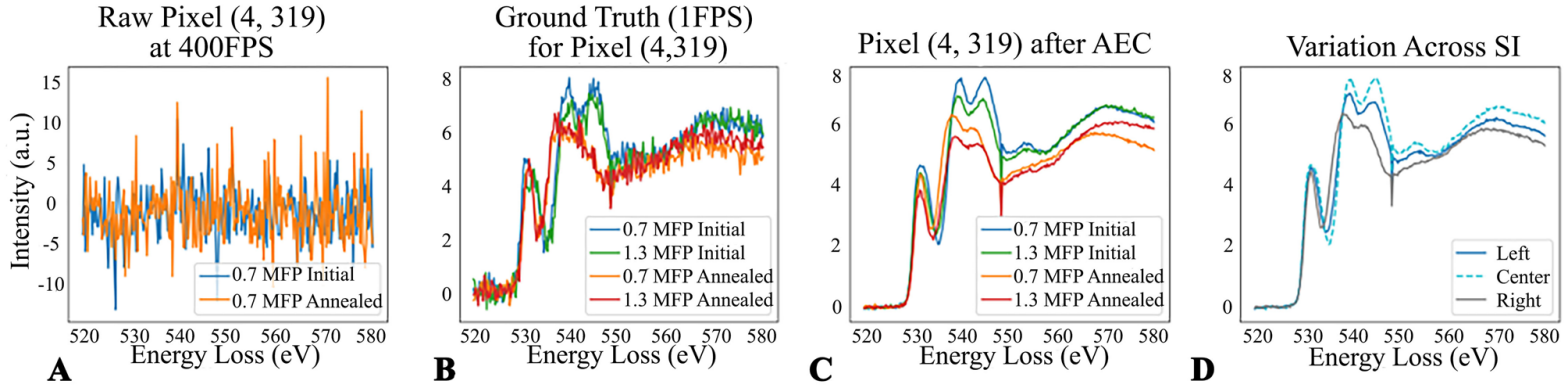
Spatial effects and fine details variation of predictions on the thin, initial state SI at 400FPS. (**A**) Comparison of the EELS data from an individual pixel (**B**) Corresponding Ground Truths for the different SI at pixel (4319) (**C**) Autoencoder denoised results on different 400FPS pixels (**D**) Comparison of reproduction of STEM probe position in the denoised results, across the 600nm SI box, for the 0.7MFP annealed sample.

Also of importance was the level of detail maintained in the denoised samples. Overall intensity trends in thickness-related variation (Fig. 2B) were maintained in the denoised 400FPS spectra (Fig. 7C). However, while translation variation from STEM probe position (Fig. 2C) were seen in the predictions (Fig. 7D), it was not as pronounced as in the ground truth spectra. This is likely do to the pooling nature of convolution minimizing variation between similar spectra and was expected due to previous applications by Chatzidakis and Botton in mitigating spectral shifts due to calibration difference^31^. Relative peak intensity, on average, was maintained in the denoised and PCA reconstruction spectra are displayed in Table 2 of the Supplemental Information.

Lastly, classification accuracy of the framework was evaluated for the 400FPS raw data and three other simulated frame rates. Using the 5-dimension latent space representation of the 400FPS input spectra, the classification algorithm achieved 82.0 percent accuracy in determining if the input spectra had oxidized. This indicates the encoder is successfully learning unique features of the low signal to noise ratio input data and is able to overcome shifts in the spectra due to probe position, as well as spectral changes due to thickness effects. When the frame rate was reduced to 200, 100, and 25 frames per second, the classification accuracy increased to 88.5 percent, 92.3 percent, and 93.0 percent respectively. Corresponding accuracy values for all SI are shown in Table 3 of the Supplemental Information.

Previous work by Chatzidakis and Botton^31^ explored convolutional and dense classifying models to overcome translational shifts due to calibration differences between machines of EELS spectra for three different electron states of manganese. While we did not achieve the same 100 percent classification accuracy for our perovskite samples, classification accuracy was a secondary goal used to explore the latent representation of the data. Nevertheless, we achieved a classification accuracy of 93.0 percent and 85.8 percent on high SNR (20fps) and very low signal to noise ratio (400FPS) data, respectively. An algorithm trained with the explicit goal of classifying the denoised spectra directly would likely achieve even high classification accuracy. Peak statistic comparisons between the ground truth spectra and denoised spectra prove that fine-detail structure is maintained in the denoised spectra. Notably, at lower frame rates, the classification algorithm begins to have difficulties with the thick annealed spectra and mis-classifies as initial state. However, at low frame rates, it would likely not be necessary to use a deep learning framework for classification.

**Figure 8:**
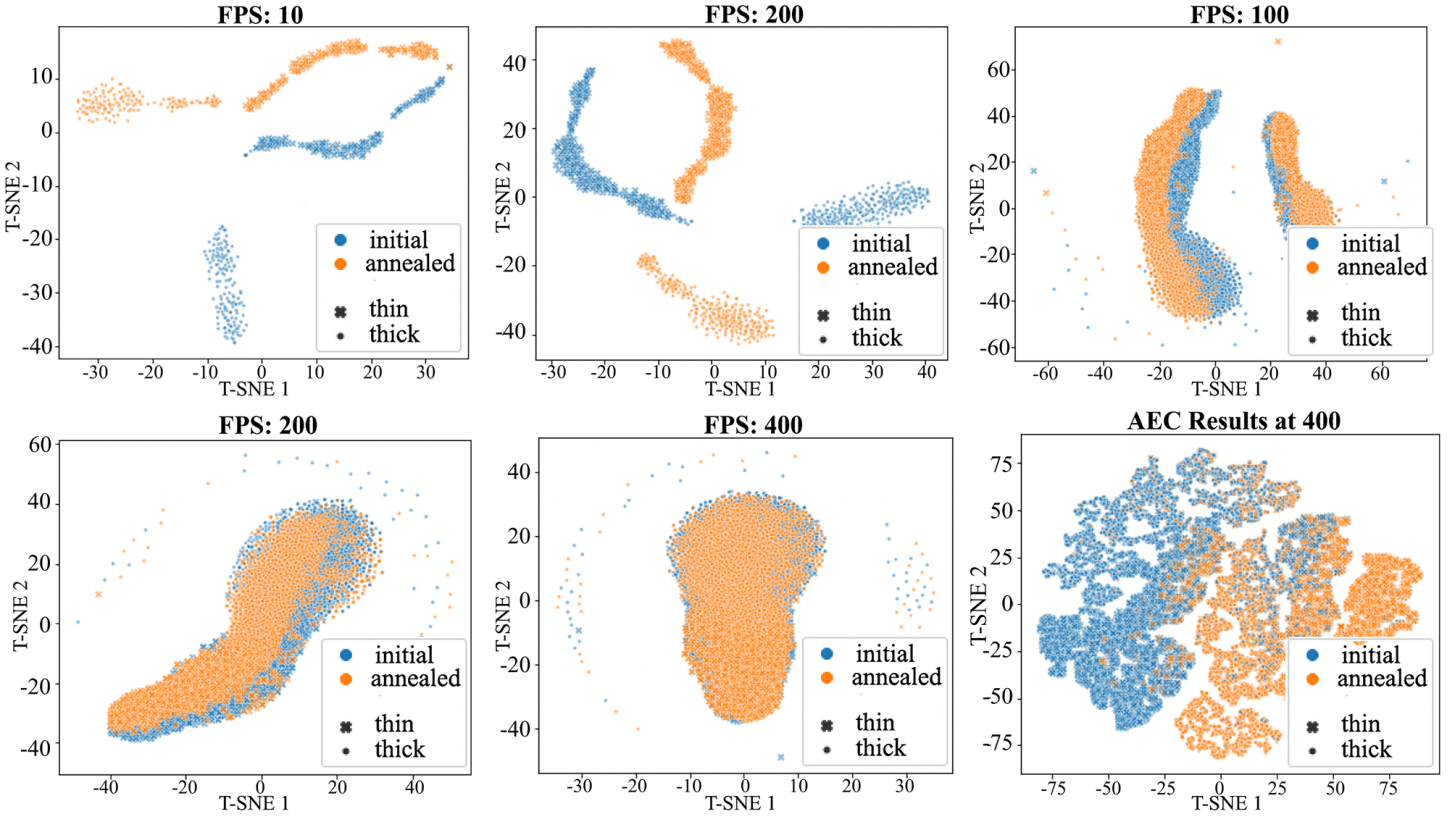
Two dimensional t-SNE plots of test spectra (raw data) at varied frame rates, including denoised 400 FPS spectra. Blue and orange markers represent the initial and annealed states, respectively. An ’X’ marker represents a thin sample and ’o’ marker represents a thick sample.

Figure 8 shows 2-D t-SNE plots for the raw spectra data at five different frame rates, plus AEC denoise spectra at 400 FPS. Below 100 FPS, it would be possible to use clustering algorithms instead of a deep learning approach to classify oxidation state and sample thickness, because distinct clusters are formed. At 100FPS and above, the 2-component t-SNE representation of annealed and initial state input spectra become increasingly intermixed. At 400FPS, the clusters are completely entangled, yet the classification algorithm is still able to achieve 85.8 percent binary classification accuracy, proving that the AEC is able to learn relevant, unique information from even the low SNR data. The denoised 400 FPS spectra t-SNE representation begins to form distinct clusters, similar to raw data at low frame rates. This is important for unsupervised and semi-supervised learning applications, as it may be possible to employ clustering algorithms to classify non-labeled data if categorical clusters can be dis-entangled. Additionally, an abundance of recent work has focused on spatial components for linear unmixing, denoising and classification of hyperspectral data, such as Bayesian mode based on Markov random fields v , image in-painting by beta-process factor analysis^33^, and Gaussian processes methods^53^. Future work aims to incorporate similar spatial components and relationships for in-situ annealed samples.

## Conclusions

Our ML approach successfully classified spectra from different thickness for samples with 85.8 percent accuracy and reduced noise enough to visually compare to reference spectra. Trends in thickness-dependent details were preserved in the auto-encoder denoised spectra for the initial state SIs. Denoised annealed spectra produced peaks that were 0.3–0.4 eV In all benchmarks, the auto-encoder denoised spectra had similar performance to the PCA reconstructions, with respect to MSE.

This paves the way for more regular use of ML codes in spectra acquisition to assist experiments in situ, and application of more complex ML, e.g., unsupervised learning, to take advantage of high frame rates and study non-equilibrium phenomena. The high classification accuracy on 400FPS latent space representations indicates the autoencoder is ‘learning’ relevant and unique low-dimensional features representing the $\hbox {SrFeO}_{{3}}$ and $\hbox {SrFeO}_{2.5}$ oxidation states. Future work will build on the foundation established here to investigate low-dimensional latent representations further for potential unsupervised applications without defined, finite classes.

## Supplementary Information

Supplementary Information.

## References

[1] Tate MW (2016). High Dynamic Range Pixel Array Detector for Scanning Transmission Electron Microscopy. Microscopy and Microanalysis.

[2] McMullan G, Faruqi AR, Henderson R (2016). Direct Electron Detectors. Methods in Enzymology.

[3] Booth, C. K2: A super-resolution electron counting direct detection camera for cryo-em. *Tech. Rep.* **S2**, 10.1017/S1431927612002243 (2012).

[4] Hart JL (2017). Direct detection electron energy-loss spectroscopy: a method to push the limits of resolution and sensitivity. Scientific Reports.

[5] Bai, X. c., McMullan, G. & Scheres, S. H. How cryo-EM is revolutionizing structural biology. Trends Biochem. Sci.**40**, 49–57, 10.1016/j.tibs.2014.10.005 (2015). 10.1016/j.tibs.2014.10.005 25544475

[6] Jiang Y (2018). Electron ptychography of 2D materials to deep sub-ångström resolution. Nature.

[7] Socher, R., Bauer, J., Manning, C. D. & Ng., A. Y. Parsing With Compositional Vector Grammars. Proceedings of ACL 2013 455–465 (2013).

[8] Sutskever, I., Vinyals, O. & Le, Q. V. Sequence to sequence learning with neural networks. CoRR arXiv:1409.3215 (2014).

[9] Hamilton, W. L., Leskovec, J. & Jurafsky, D. Diachronic word embeddings reveal statistical laws of semantic change. CoRR arXiv:1605.09096 (2016).

[10] Li, Q. et al. Medical image classification with convolutional neural network. In 2014 13th International Conference on Control Automation Robotics and Vision, ICARCV 2014, 844–848, 10.1109/ICARCV.2014.7064414 (Institute of Electrical and Electronics Engineers Inc., 2014).

[11] Kanellopoulos I, Wilkinson GG (1997). Strategies and best practice for neural network image classification. International Journal of Remote Sensing.

[12] Sattlecker M, Bessant C, Smith J, Stone N (2010). Investigation of support vector machines and Raman spectroscopy for lymph node diagnostics. Analyst.

[13] Giacinto G, Roli F (2001). Design of effective neural network ensembles for image classification purposes. Image and Vision Computing.

[14] Xie J, Xu L, Chen E (2012). Image denoising and inpainting with deep neural networks. Advances in neural information processing systems.

[15] Cho, K. Boltzmann machines and denoising autoencoders for image denoising. arXiv:1301.3468 (2013).

[16] Vincent, P., Larochelle, H., Bengio, Y. & Manzagol, P.-A. Extracting and composing robust features with denoising autoencoders. In* Proceedings of the 25th international conference on Machine learning*, 1096–1103 (2008).

[17] Vincent, P. *et al.* Stacked denoising autoencoders: Learning useful representations in a deep network with a local denoising criterion. *J. Mach. Learn. Res.* **11**, (2010).

[18] Jain V, Seung S (2008). Natural image denoising with convolutional networks. Advances in neural information processing systems.

[19] Gondara, L. Medical image denoising using convolutional denoising autoencoders. In* 2016 IEEE 16th International Conference on Data Mining Workshops (ICDMW)*, 241–246, 10.1109/ICDMW.2016.0041 (2016).

[20] Kwiatkowski A, Gnyba M, Smulko J, Wierzba P (2010). Algorithms of chemicals detection using Raman spectra. Metrology and Measurement Systems.

[21] Carey C, Boucher T, Mahadevan S, Bartholomew P, Dyar MD (2015). Machine learning tools for mineral recognition and classification from Raman spectroscopy. Journal of Raman Spectroscopy.

[22] Yedra L (2014). Oxide wizard: An EELS application to characterize the white lines of transition metal edges. Microscopy and Microanalysis.

[23] Roels J (2020). An interactive imagej plugin for semi-automated image denoising in electron microscopy. Nature Communications.

[24] Wang F, Henninen TR, Keller D, Erni R (2020). Noise2atom: unsupervised denoising for scanning transmission electron microscopy images. Applied Microscopy.

[25] Lin R, Zhang R, Wang C, Yang X-Q, Xin HL (2021). Temimagenet training library and atomsegnet deep-learning models for high-precision atom segmentation, localization, denoising, and deblurring of atomic-resolution images. Scientific Reports.

[26] Zhang, C., Han, R., Zhang, A. R. & Voyles, P. Denoising atomic resolution 4d scanning transmission electron microscopy data with tensor singular value decomposition. *Ultramicroscopy* **219**, 113123. 10.1016/j.ultramic.2020.113123 (2020). 10.1016/j.ultramic.2020.113123 33032160

[27] Tan H, Verbeeck J, Abakumov A, Van Tendeloo G (2012). Oxidation state and chemical shift investigation in transition metal oxides by EELS. Ultramicroscopy.

[28] Zhang S, Livi KJ, Gaillot AC, Stone AT, Veblen DR (2010). Determination of manganese valence states in (Mn3+, Mn 4+) minerals by electron energy-loss spectroscopy. American Mineralogist.

[29] Kalinin, S. V. et al. Separating physically distinct mechanisms in complex infrared plasmonic nanostructures via machine learning enhanced electron energy loss spectroscopy. arXiv:2009.08501 [cond-mat] (2020). .

[30] Blum, T. *et al.* Machine learning method reveals hidden strong metal-support interaction in microscopy datasets. *Small Methods n/a* **2100035**, 10.1002/smtd.202100035 (2021). 10.1002/smtd.202100035 34928097

[31] Chatzidakis M, Botton GA (2019). Towards calibration-invariant spectroscopy using deep learning. Scientific Reports.

[32] Muller DA (2008). Atomic-scale chemical imaging of composition and bonding by aberration-corrected microscopy. Science.

[33] Stevens A, Yang H, Carin L, Arslan I, Browning ND (2014). The potential for bayesian compressive sensing to significantly reduce electron dose in high-resolution STEM images. Microscopy.

[34] Pan M, Crozier PA (1993). Low-dose high-resolution electron microscopy of zeolite materials with a slow-scan CCD camera. Ultramicroscopy.

[35] Fujiyoshi Y (1998). The structural study of membrane proteins by electron crystallography. Advances in Biophysics.

[36] Xie, Y. J. *et al.* Electronic phase diagram of epitaxial La1-xSr xFeO3 films. *Appl. Phys. Lett.* **105**, 062110. 10.1063/1.4893139 (2014).

[37] Lefler, B. M. *et al.* Reconfigurable lateral anionic heterostructures in oxide thin films via lithographically defined topochemistry. *Phys. Rev. Mater.* **3**, 073802. 10.1103/PhysRevMaterials.3.073802 (2019).

[38] Khare A (2017). Topotactic Metal-Insulator Transition in Epitaxial SrFeO_*x*_ Thin Films. Advanced Materials.

[39] Saleem, M. S. et al. Electric Field Control of Phase Transition and Tunable Resistive Switching in SrFeO 2.5.* ACS App. Mater. Interfaces* **11**, 6581–6588, 10.1021/acsami.8b18251 (2019). 10.1021/acsami.8b18251 30663876

[40] de la Peña, F. et al. hyperspy: Hyperspy 1.0.1, 10.5281/zenodo.58841 (2016).

[41] Bishop CM (1995). Training with noise is equivalent to tikhonov regularization. Neural computation.

[42] An G (1996). The effects of adding noise during backpropagation training on a generalization performance. Neural Computation.

[43] Meng L, Ding S, Xue Y (2017). Research on denoising sparse autoencoder. International Journal of Machine Learning and Cybernetics.

[44] Yakovlev S, Balsara NP, Downing KH (2012). Limits of spatial and compositional resolution of electron energy loss spectroscopy of soft materials. Ultramicroscopy.

[45] Srivastava N, Hinton G, Krizhevsky A, Sutskever I, Salakhutdinov R (2014). Dropout: A simple way to prevent neural networks from overfitting. Journal of Machine Learning Research.

[46] Srivastava, N. Improving neural networks with dropout (2013).

[47] Liang, J. & Liu, R. Stacked denoising autoencoder and dropout together to prevent overfitting in deep neural network. In* 2015 8th International Congress on Image and Signal Processing (CISP)*, 697–701 (2015).

[48] Poole, B., Sohl-Dickstein, J. & Ganguli, S. Analyzing noise in autoencoders and deep networks. arXiv:1406.1831 (2014).

[49] Mao, X., Shen, C. & Yang, Y. Image restoration using convolutional auto-encoders with symmetric skip connections. CoRR arXiv:1606.08921 (2016).

[50] Chai T, Draxler RR (2014). Root mean square error (rmse) or mean absolute error (mae)?-arguments against avoiding rmse in the literature. Geoscientific model development.

[51] Murali Mohan Babu, Y. PCA based image denoising. signal & image processing. Int. J. **3**, 236–244, 10.5121/sipij.2012.3218 (2012).

[52] Salmon J, Harmany Z, Deledalle CA, Willett R (2014). Poisson noise reduction with non-local PCA. Journal of Mathematical Imaging and Vision.

[53] Kalinin, S., Lupini, A. R., Vasudevan, R. & Ziatdinov, M. Gaussian process analysis of electron energy loss spectroscopy (eels) data: parallel reconstruction and kernel control. Computational Physics (2020).

