# Supplementary material for: RapidEELS: machine learning for denoising and classification in rapid acquisition electron energy loss spectroscopy
Source: Sci Rep. 2021 Sep 30;11:19515. doi: 10.1038/s41598-021-97668-8 (PMC8484590; doi:10.1038/s41598-021-97668-8)
Supplement: Supplementary file 1 — Supplementary Information. [file 41598_2021_97668_MOESM1_ESM.pdf]

Supplemental Information for RapidEELS: Machine Learning for Denoising and Classification in Rapid Acquisition Electron Energy Loss Spectroscopy

Cassandra M. Pate[1], **James L. Hart**[1,2], **and Mitra L. Taheri**[1,*]

[1]Department of Materials Science & Engineering, Johns Hopkins University, Baltimore, MD 21218
[2]Currently at Department of Mechanical Engineering & Materials Science, Yale University, New Haven, CT 06511
[*]Corresponding Author, mtaheri4@jhu.edu

Supplemental Information

Table 1. Detailed description of layers in the neural network framework.

Model Section	Layer (type)	Kernel Size	Filter Size	Stride Size	Output Shape	Activation
Encoder	InputLayer	-	-	-	(None, 240, 1)	-
	Conv1D	8	7	2	(None, 120, 8)	ReLU
	Dropout (20%)	-	-	-	(None, 120, 8)	-
	Conv1D	16	7	2	(None, 60, 16)	ReLU
	Dropout (20%)	-	-	-	(None, 60, 16)	-
	Conv1D	16	5	2	(None, 30, 16)	ReLU
	Dropout (20%)	-	-	-	(None, 30, 16)	-
	Conv1D	32	5	2	(None, 15, 32)	ReLU
	Dropout (20%)	-	-	-	(None, 15, 32)	-
	Conv1D	64	3	2	(None, 8, 64)	ReLU
	Flatten	-	-	-	(None, 512)	-
Latent Space	Dense	-	-	-	(None, 5)	ReLU
Decoder	Dense	-	-	-	(None, 512)	-
	Reshape	-	-	-	(None, 8, 64)	-
	Conv1DTranspose	64	3	2	(None, 15, 64)	ReLU
	Conv1DTranspose	32	5	2	(None, 30, 32)	ReLU
	Conv1DTranspose	16	5	2	(None, 60, 16)	ReLU
	Conv1DTranspose	16	7	2	(None, 120, 16)	ReLU
	Conv1DTranspose	8	7	2	(None, 240, 8)	ReLU
	Conv1DTranspose	1	1	1	(None, 240, 1)	Linear
Classifier	InputLayer	-	-	-	(None, 240, 1)	-
	Encoder Model	(see above)			(None, 5)	-
	Dense	-	-	-	(None, 2)	Softmax

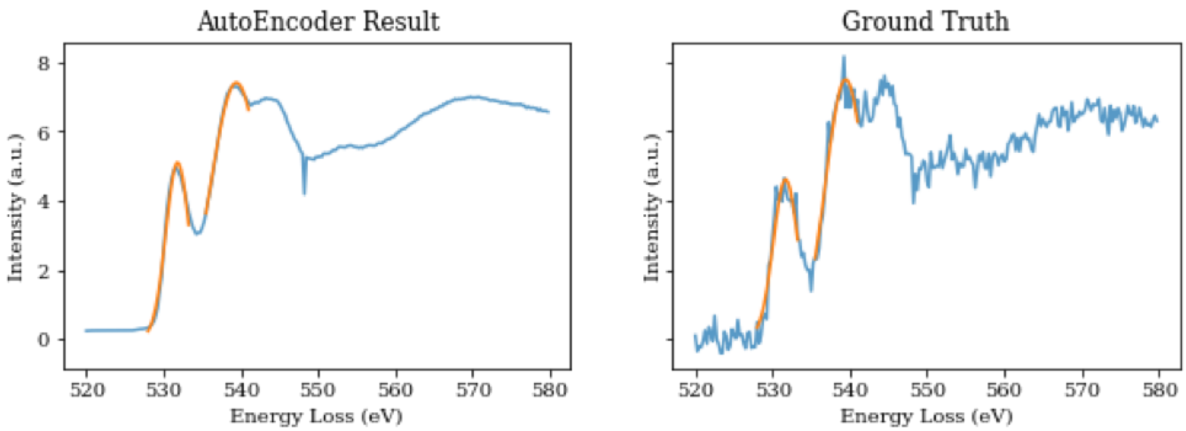

Figure 1. Example of Gaussian fit for one pixel denoised by the Autoendcoder and the corresponding ground truth spectrum from training the neural network.

Table 2. Variation from Ground Truth of peak characteristics for Auto-encoder denoised spectra and PCA reconstruction. Red indicates a value outside of Ground Truth range, including error. Peak position is measure as change in distance (eV) between pre-peak and Oxygen K-edge peak.

		AEC Results on 400FPS		1 FPS Ground Truths		3 Comp. PCA Reconstrct.	
Sample Thickness	Oxidation State	ΔPeak Positions (eV)	ΔPeak Heights (Intensity)	ΔPeak Positions (eV)	ΔPeak Heights (Intensity)	ΔPeak Positions (eV)	ΔPeak Heights (Intensity)
0.65-0.80MFP ("Thin")	Initial	7.43 ± 0.16	2.38 ± 0.15	7.59 ± 0.21	2.54 ± 0.20	7.56 ± 0.22	2.55 ± 0.29
	Annealed	6.90 ± 0.31	2.04 ± 0.14	6.56 ± 0.17	1.68 ± 0.15	6.51 ± 0.27	1.75 ± 0.30
0.83-1.33MFP ("Thick")	Initial	7.30 ± 0.16	2.31 ± 0.15	7.39 ± 0.18	2.54 ± 0.19	7.41 ± 0.28	2.59 ± 0.17
	Annealed	6.97 ± 0.24	2.06 ± 0.09	6.36 ± 0.28	1.85 ± 0.22	6.36 ± 0.39	1.91 ± 0.15

Table 3. Classification accuracy at statistics for different frame rate spectra by weighted average of all Sis at corresponding frame rate, thickness and oxidation state.

	AEC Denoised Classification Accuracy				
	400 FPS	200 FPS	100 FPS	20 FPS	1 FPS
**Entire SI**	0.858	0.918	0.944	0.930	0.904
**Thin Initial**	0.871	0.947	0.980	1.000	1.000
**Thick Initial**	0.881	0.929	0.987	0.996	1.000
**Thin Annealed**	0.859	0.886	0.889	0.908	1.000
**Thick Annealed**	0.820	0.909	0.918	0.815	0.615
